# Construction of Predictive Machine Learning Model of Glioma‐Associated Gut Microbiota

**DOI:** 10.1002/brb3.70843

**Published:** 2025-09-09

**Authors:** Ze Li, Kai Zhao, Hongyu Liu, Jialin Liu, Xu Chen, Wentao Hu, Er Wen, Kai Zhang, Ling Chen

**Affiliations:** ^1^ Department of Neurosurgery First Medical Center of the Chinese PLA General Hospital Beijing People's Republic of China; ^2^ China Medical University Shenyang People's Republic of China; ^3^ Department of Neurosurgery Beijing Tsinghua Changgung Hospital, Tsinghua University Beijing People's Republic of China

**Keywords:** glioma, gut microbiome, metagenomic sequencing, machine learning models

## Abstract

**Background:**

The gut microbiota plays a crucial role in the development of glioma. With the evolution of artificial intelligence technology, applying AI to analyze the vast amount of data from the gut microbiome indicates the potential that artificial intelligence and computational biology hold in transforming medical diagnostics and personalized medicine.

**Methods:**

We conducted metagenomic sequencing on stool samples from 42 patients diagnosed with glioma after operation and 30 non‐intracranial tumor patients and developed a Gradient Boosting Machine (GBM) machine learning model to predict the glioma patients based on the gut microbiome data.

**Results:**

The AUC‐ROC for the GBM model was 0.79, indicating a good level of discriminative ability.

**Conclusions:**

This method's efficacy in discriminating between glioma cells and normal controls underscores the potential of machine learning models in leveraging large datasets for clinical insights.

## 1 Introduction

Gliomas, prevalent malignant central nervous system tumors (Aldape et al. 2019; Molinaro et al. 2019), exhibit complex pathology that contributes to their aggressiveness, rapid progression, and high recurrence rates, complicating treatment (Louis et al. 2007). Current guidelines prioritize surgical interventions as the primary treatment modality for gliomas, supplemented by traditional therapies such as radiation and chemotherapy, as well as emerging treatments like electric field and immunotherapy (Ballo et al. 2023; Yasinjan et al. 2023). However, the median survival period remains low, with significant individual variability. Forward‐looking prediction has always been a focal point in glioma research. With the advancement in tumor studies, the tumor microenvironment and the interaction between the tumor and its host have become hot topics in contemporary glioma research (Dono et al. 2022; Nejman et al. 2020). The gut microbiome significantly influences tumor oncogenesis and development, potentially revolutionizing glioma diagnosis and treatment (Aljarrah et al. 2024; W. Wang et al. 2024; Liang et al. 2022).

The gut microbiome, the body's largest microbial community located in the intestines, significantly influences host metabolism, immunity, cognition, and disease pathology (Cresci and Bawden 2015; Denny et al. 2016). Research on animals has validated the strong link between the gut microbiome and gliomas (Dono et al. 2022; Fan et al. 2022). As sequencing technologies have rapidly advanced, traditional 16S RNA sequencing, limited by its sequencing principle, fails to distinguish at lower taxonomic levels. In contrast, shotgun metagenomic sequencing, which sequences all genomic DNA in a single sample, can achieve sequencing depth at the species level. Therefore, the sequencing results produce a vast amount of data, and how to effectively utilize this type of data remains a challenge in current research. Previous studies have mostly limited themselves to simple comparisons of the gut microbial abundance between glioma patients and healthy populations or to drug‐related experiments in animal models. With the evolution of artificial intelligence technology, applying AI to analyze the vast amount of data from the gut microbiome has become one of the current challenges and hot topics. This article utilizes various computational methods to analyze gut gene data from glioma patients, aiming to establish an accurate computational learning model for forward‐looking prediction of glioma onset.

## 2 Materials and Methods

### 2.1 Data Collection

Following approval from the Chinese PLA General Hospital and obtaining patient consent, stool samples were collected from 79 patients, comprising 49 with intracranial tumors and 30 control patients without intracranial tumors. The fresh feces were gathered before the operation using sterile tools and then placed in sterile sampling tubes with ice packs for transport to the lab. Samples designated for shotgun metagenomic sequencing were stored at −80°C until processing. Postoperatively, 42 intracranial tumor patients were diagnosed with glioma. Ultimately, 72 stool samples qualified for shotgun metagenomic sequencing.

We collected demographic and clinicopathological data, such as gender, age, and tumor grade, from the medical records of the 72 patients after their initial hospital admission. This study involved analyzing shotgun metagenomic sequencing data from 72 stool samples.

The sample collection included participants who met the following criteria: the study included glioma patients with confirmed pathological staging post‐surgery, as per the Guidelines for Diagnosis of Gliomas, and control group patients with normal brain MRI results. Participants had no history of other malignancies, intestinal diseases, prior antitumor treatments, or recent antibiotic or intestinal microbiological use within a month before enrollment. In addition, they had no history of consciousness injury or cognitive dysfunction.

We collected fecal samples from the patients on the first day after their admission and provided instructions to retain the middle portion of the feces while avoiding urine contamination when using a sterile fecal collection tube. The collected fecal specimens were then divided into 2 mL EP tubes (200 mg/tube) and stored in sterile ice boxes at −80°C for freezing storage.

### 2.2 DNA Extraction

Total genomic DNA was extracted from 0.5 g of stool using the FastPure Stool DNA Isolation Kit (Magnetic bead) (MJYH, Shanghai, China) following the manufacturer's instructions. Concentration and purity of extracted DNA were determined with SynergyHTX and NanoDrop2000, respectively. DNA quality was assessed using a 1% agarose gel.

### 2.3 Metagenomic Sequencing

The DNA extract was fragmented to an average size of approximately 400 bp using the Covaris M220 (Gene Company Limited, China) for paired‐end library construction. A paired‐end library was constructed using NEXTFLEX Rapid DNA‐Seq (Bioo Scientific, Austin, TX, USA) and sequenced on an Illumina NovaSeq X Plus (Illumina Inc., San Diego, CA, USA) at Majorbio Bio‐Pharm Technology Co. Ltd. (Shanghai, China) with the NovaSeq X Series 25B Reagent Kit, following the manufacturer's instructions.

### 2.4 Model Training and Evaluation

The dataset included samples from normal subjects as well as patients with various types of gliomas, including GBM. Stool samples were collected and processed to extract DNA, followed by shotgun metagenomic sequencing to identify and quantify microbial taxa. At the genus level, taxa representing less than 0.01% of total reads were excluded, resulting in 947 features for analysis. The dataset was divided into training (75%) and validation (25%) sets, and a 10‐fold cross‐validation approach was used. Features with low variance were removed to reduce noise. The Gradient Boosting Machine (GBM) algorithm was selected for its ability to handle complex, nonlinear relationships. Grid search and cross‐validation were employed to optimize hyperparameters.

The GBM model was trained on the training dataset, with feature importance evaluated to prune less informative features. During each iteration of the 10‐fold cross‐validation, the model was fitted on training subsets and evaluated on validation subsets. The model used 300 estimators (*n*_estimators = 300), no depth limit (x_depth: none), and a random state of seven (random_state = 7). The training‐to‐test set ratio was 3:1, with 10‐fold cross‐validation during training. The model's performance was assessed using accuracy, precision, recall, and the F1 score. These metrics offered a thorough assessment of the model's accuracy in identifying glioma patients. The study indicated that gut microbiome profiles could be utilized in noninvasive diagnostic tools for glioblastoma, emphasizing the link between the gut microbiome and brain health.

## 3 Result

### 3.1 Clinical Characteristics

The study included 72 patients, as outlined in Table 1 . The cohort included 42 glioma patients and 30 control subjects. The baseline data, including age, sex, and BMI score, were well balanced and comparable, as no statistically significant differences were observed (*p* > 0.05).

**TABLE 1:** Characteristics of the 42 patients with glioma and 30 controls.

Characteristics	Glioma patients, *n* (%) (*n* = 42)	Controls, *n* (%) (*n* = 30)	*p* value
**Gender**			
Male	26 (61.90%)	12 (40%)	0.066
Female	16 (38.10%)	18 (60%)	0.066
Age	47.45±13.81	52.60±10.76	0.08
BMI	24.64±3.64	25.60±3.23	0.242
Smoking	16 (28.10%)	18 (60%)	0.066
Drinking	23 (54.76%)	0	8.95828E−07
Hypertension	2 (4.76%)	5 (16.67%)	0.201
Grade			
II	14 (33.33%)	—	—
III	10 (23.81%)	—	—
IV	18 (42.86%)	—	—
Histological type		—	—
Oligodendrocytoma	7 (16.67%)	—	—
Astrocytoma	3 (7.14%)	—	—
Diffuse astrocytoma	6 (14.29%)	—	—
Pleomorphic	2 (4.76%)	—	—
Anaplastic astrocytoma	6 (14.29%)	—	—
Glioblastoma	18 (42.86%)	—	—

**TABLE 2:** Predicting the results of the glioma GBM model.

	Precision	Recall	*F*1‐score	Support
1	1.00	0.67	0.80	12
0	0.60	1.00	0.75	6

### 3.2 Model Performances

**FIGURE 1:**
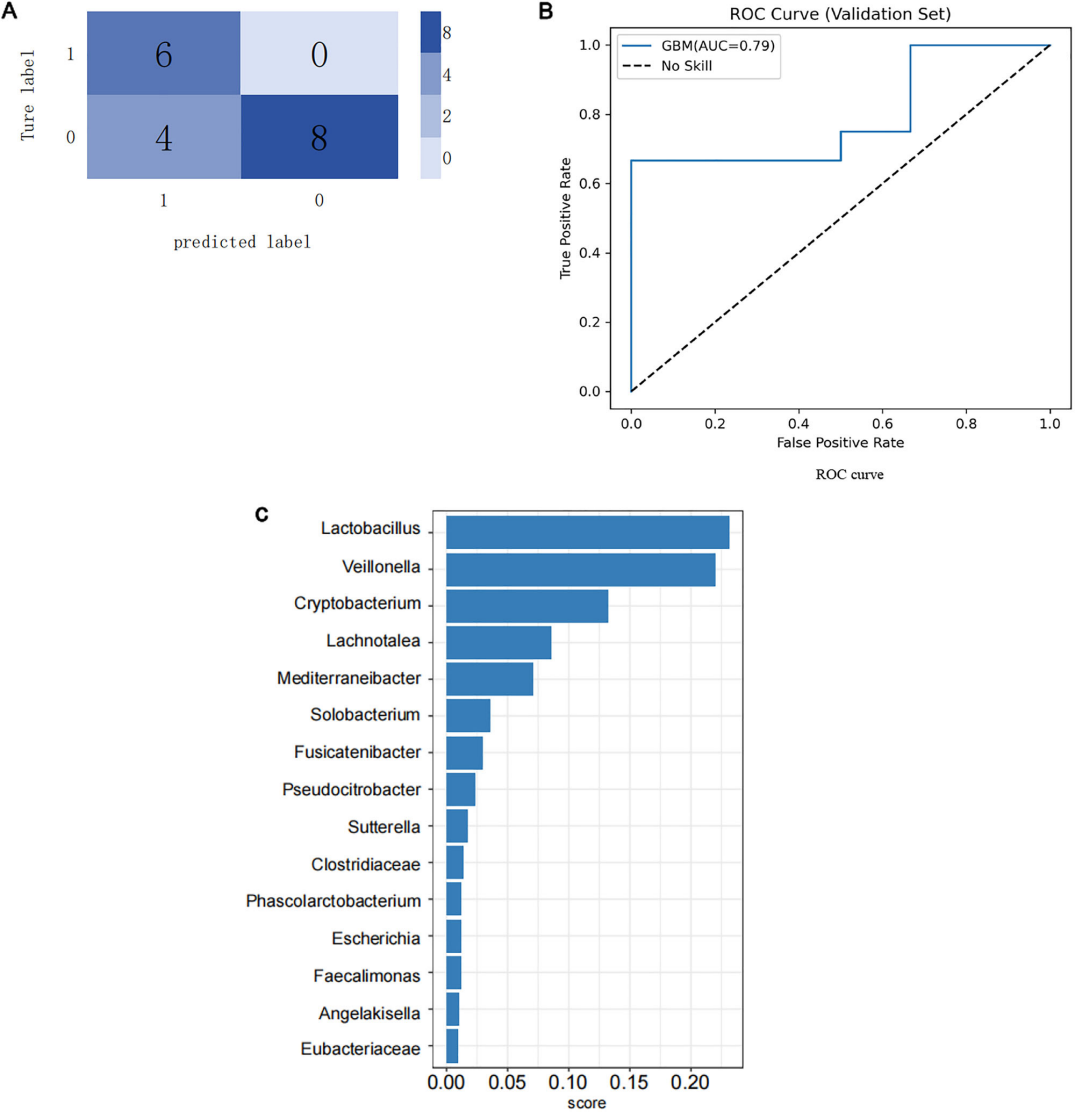
GBM machine learning model for predicting glioma‐based on the gut microbiota. From the validation sets, (A) the confusion matrix in the training set of the GBM model. The horizontal axis denotes the predicted labels of the model, and the vertical axis indicates the actual status of the samples. The values “1” and “0” represent positive and negative predictions, respectively. The numbers inside various boxes indicate the sample count. Color depth increases with the number of samples, resulting in a darker appearance as sample quantity rises. (B) ROC curves for the GBM model's training and validation sets. The horizontal axis represents the model's false positive rate in predicting glioma, while the vertical axis indicates the true positive rate. The AUC value represents the area under the curve, where a higher AUC indicates greater model prediction accuracy. (C) Top 10 gut microbiotas with high importance in the gradient boosting machine‐based classifier.

The GBM model developed in this study demonstrated a notable ability to predict glioma status based on the gut microbiome profiles of subjects, as shown in Table 2, which is the predicted result of the model from validation sets. The model's performance was primarily evaluated using the area under the receiver operating characteristic curve (AUC‐ROC) metric. The AUC‐ROC for the GBM model from validation sets was 0.79 (Figure 1), indicating a good level of discriminative ability.

To explore the contribution of gut microbiota to the classification process, Figure 1C presents the top 10 gut microbiota with the highest importance scores. Among these, *Lactococcus* ranks as the most significant feature in the GBM model, underscoring its critical role in predicting glioma onset.

## 4 Discussion

This study highlights the growing importance of the gut microbiome in glioma pathophysiology, shifting focus from its traditional association with gastrointestinal health. Our study confirms the intricate relationship between the gut microbiome and the central nervous system and underscores the potential of using gut microbiome data for noninvasive glioma diagnostics.

The gut microbiome, often termed the host's “second genome” (Thursby 2017), plays a crucial role in host metabolism, immunity, cognition, and neurological disease progression. Its dynamic interaction with the host offers new insights and potential treatments for gliomas, surpassing traditional methods that have struggled to significantly improve median survival rates for patients.

Significantly, our application of the GBM algorithm to analyze gut microbiome data for glioma patients represents an innovative approach in the predictive diagnostics of gliomas. This method's efficacy in discriminating between glioma cells and normal controls underscores the potential of machine learning models in leveraging large datasets for clinical insights. Notably, the distinction drawn by the GBM algorithm between glioma patients and healthy controls based on gut microbiome data illustrates the profound impact of microbial compositions on glioma pathogenesis.

The rapid advancements in sequencing technologies, particularly shotgun metagenomic sequencing, have provided unprecedented depth and breadth in understanding the microbial genome (Tas et al. 2021). However, the vast amount of data generated presents both a challenge and an opportunity. Our study navigates these complexities by employing a machine learning approach to distill meaningful patterns that could aid in the early detection and treatment of gliomas. This approach is indicative of the potential that artificial intelligence and computational biology hold in transforming medical diagnostics and personalized medicine.

Our present study shows that *Lactobacillus* may play a critical role in predicting glioma onset according to the highest rank in the GBM model. The relationship between *Lactobacillus* and glioma remains complex and multifaceted, as evidenced by its dual roles in both tumor suppression and potential tumor promotion. On one hand, a recent study shows that there are significant differences in *Lactobacillus* between the healthy group and the disease group, which may be validated with (R)‐elagolix in animal experiments (C. Wang et al. 2025). This is similar to our study. Besides, studies have demonstrated that the combined administration of *Lactobacillus* and *Bifidobacterium* can inhibit glioma growth by suppressing the PI3K/AKT signaling pathway, repairing intestinal barrier damage, and modulating gut microbiota. This suggests that *Lactobacillus* may hold therapeutic potential in glioma treatment (L. Wang et al. 2022). On the other hand, certain species of *Lactobacillus*, such as *Lactobacillus plantarum*, have been observed to exhibit higher abundance in glioma patients compared to healthy individuals, raising concerns about their possible tumor‐promoting effects (Li et al. 2021). Additionally, fluctuations in the abundance of *Lactobacillus* during glioma progression and its stability following chemotherapy with temozolomide (TMZ) suggest a dynamic interaction between gut microbiota and glioma development (Amiri et al. 2021). Taken together, these findings highlight the need for further research to elucidate the precise mechanisms underlying *Lactobacillus*'s effects on glioma and to determine its safety and efficacy as a potential therapeutic strategy.

Furthermore, the lack of significant microbial changes observed in some studies, including ours, when comparing glioma patients to healthy controls, may reflect the limited number of cases studied or the variability in microbial composition among individuals. This underscores the necessity for larger, more comprehensive studies to validate the findings and explore the nuances of gut microbiome diversity and its implications for glioma diagnostics and therapeutics.

In summary, this study lays the groundwork for future investigations into the gut microbiome's involvement in glioma and highlights the promise of computational techniques in discovering new biomarkers for glioma diagnosis. The convergence of microbiology, genomics, and artificial intelligence offers promising pathways for understanding complex diseases like gliomas, potentially leading to breakthroughs in noninvasive diagnostics and personalized treatment strategies. Further research in this direction is essential to unravel the full potential of the gut microbiome in glioma pathogenesis and therapy.

## Author Contributions

Z.L., K.Z., and H.Y.L. carried out the model building and drafted the article. J.L.L., X.C., W.T.H., and E.W. performed the specimen collection. K.Z. and L.C. conceived and supervised the study. The final manuscript has been read and approved by all authors.

## Ethics Statement

Approval for the study was obtained from the relevant ethics committee, and informed consent was secured from all participants.

## Conflicts of Interest

The authors declare no conflicts of interest.

## Peer Review

The peer review history for this article is available at https://publons.com/publon/10.1002/brb3.70843.

## Data Availability

The datasets referenced in the manuscript and supplementary files can be accessed by contacting the corresponding author with a reasonable request.
